# NON-PHARMACOLOGICAL DEMENTIA CARE APPROACHES: IMPLEMENTING STAR-VA IN A SHORT-STAY, SUBACUTE MEDICAL SETTING

**DOI:** 10.1093/geroni/igae098.4245

**Published:** 2024-12-31

**Authors:** Jennifer Christopher, Theresa Christopher, Thomas Yuelling

**Affiliations:** Jesse Brown VA Medical Center, Chicago, Illinois, United States; North Central College, Naperville, Illinois, United States; Jesse Brown VA Medical Center, Chicago, Illinois, United States

## Abstract

Staff Training in Assisted Living Residences for Veterans Affairs Medical Centers (STAR-VA) is an evidenced-based, interdisciplinary, non-pharmacological approach to managing dementia-related behavioral and psychological symptoms in long-term care settings (Karlin et al., 2017). The program involves a psychologist and nurse who provide dementia education, facilitate behavioral analysis, and implement person-centered behavior plans. This study highlights the piloting of STAR-VA in a short-stay, subacute, medical rehabilitation setting. The case study involves a 74-year-old African American, male, Veteran diagnosed with Dementia due to Multiple Etiologies (Frontotemporal Degeneration and Vascular Disease). The Veteran exhibited elopement attempts, refusal to follow directions, falls, verbal aggression, and emotional distress. Behavioral analysis suggested these were related to lack of independence, boredom, psychosocial stressors, and medication side effects. Behavior plan interventions included individual psychotherapy, scheduled recreation therapy sessions focused on preferred pleasant activities, and chaplain services. The clinical pharmacist reviewed the medication list and psychiatry and general medicine providers adjusted medications. Care providers were encouraged to use effective nonverbal and verbal communication strategies, including the “listen with respect, comfort, and redirect” approach. After two months, assessments (i.e., Neuropsychiatric Inventory Questionnaire, Cohen-Mansfield Agitation Inventory–Short Form, Geriatric Depression Scale–Short Form) revealed discontinued elopement attempts, compliance with care, improved mood, reduced falls, and decreased nursing staff distress. These interventions facilitated the Veteran’s transition to long-term community placement. Six-month follow-up assessments revealed the Veteran remained behaviorally stable. This case demonstrated that STAR-VA can be effectively adapted for short-stay, subacute medical rehabilitation settings. It further supports interdisciplinary, non-pharmacological approaches for dementia.

